# Risk Prediction of Barrett’s Esophagus in a Taiwanese Health Examination Center Based on Regression Models

**DOI:** 10.3390/ijerph18105332

**Published:** 2021-05-17

**Authors:** Po-Hsiang Lin, Jer-Guang Hsieh, Hsien-Chung Yu, Jyh-Horng Jeng, Chiao-Lin Hsu, Chien-Hua Chen, Pin-Chieh Wu

**Affiliations:** 1 Department of Emergency Medicine, Kaohsiung Veterans General Hospital, Kaohsiung 813, Taiwan; phlin@vghks.gov.tw; 2 Department of Electrical Engineering, I-Shou University, Kaohsiung 840, Taiwan; jghsieh@isu.edu.tw (J.-G.H.); hua751016@gmail.com (C.-H.C.); 3 Division of Gastroenterology and Hepatology, Department of Internal Medicine, Kaohsiung Veterans General Hospital, Kaohsiung 813, Taiwan; hcyu@vghks.gov.tw; 4 Health Management Center, Kaohsiung Veterans General Hospital, 386, Ta-Chung 1st Road, Kaohsiung 813, Taiwan; clhsu@vghks.gov.tw; 5 Institute of Health Care Management, Department of Business Management, National Sun Yat-sen University, Kaohsiung 804, Taiwan; 6 Department of Nursing, Meiho University, Pingtung 912, Taiwan; 7 Department of Information Engineering, I-Shou University, Kaohsiung 840, Taiwan; jjeng@isu.edu.tw; 8 Department of Emergency Medicine, Taichung Veterans General Hospital Chiayi Branch, Chia-Yi 600, Taiwan; 9 Department of Chemical Engineering and Institute of Biotechnology and Chemical Engineering, I-Shou University, Kaohsiung 840, Taiwan

**Keywords:** Barrett’s esophagus, logistic models, neural networks, computer, Taiwan

## Abstract

Determining the target population for the screening of Barrett’s esophagus (BE), a precancerous condition of esophageal adenocarcinoma, remains a challenge in Asia. The aim of our study was to develop risk prediction models for BE using logistic regression (LR) and artificial neural network (ANN) methods. Their predictive performances were compared. We retrospectively analyzed 9646 adults aged ≥20 years undergoing upper gastrointestinal endoscopy at a health examinations center in Taiwan. Evaluated by using 10-fold cross-validation, both models exhibited good discriminative power, with comparable area under curve (AUC) for the LR and ANN models (Both AUC were 0.702). Our risk prediction models for BE were developed from individuals with or without clinical indications of upper gastrointestinal endoscopy. The models have the potential to serve as a practical tool for identifying high-risk individuals of BE among the general population for endoscopic screening.

## 1. Introduction

Esophageal adenocarcinoma (EAC) has a poor prognosis, with a 5-year survival rate of <15% [1]. Over the last three decades, the incidence of EAC has risen sharply in many countries around the world [2]. In the United States, the incidence of EAC increased from 0.40 cases per 100,000 individuals in 1975 to 2.58 cases per 100,000 individuals in 2009, a more than 6-fold increase [3]. Upward trends in the incidence of EAC have also been observed in Asian countries [4,5]. Barrett’s esophagus (BE) is a well-documented precancerous condition of EAC [1]. Although the prevalence of BE has been lower in Asian populations than in Western populations, the rate has increased from 0.8% in 1991–1999 to 2.2% in 2010–2014 [6]. A study reported that BE is probably under diagnosed in most parts of Asia [7], which may affect the prognosis of EAC. A recent meta-analysis demonstrated that the development of endoscopic therapy for BE and early-stage EAC, targeted BE screening, and endoscopic surveillance of patients with BE could provide better survival outcomes [8].

For now, the diagnostic tools for BE are upper gastrointestinal (UGI) endoscopy and histological confirmation [9,10]. Due to their invasiveness and cost, however, the methods’ usefulness in BE screening is limited. Identifying target populations for BE screening therefore remains a challenge. The current BE screening guidelines recommend that patients with gastroesophageal reflux disease (GERD) with several risk factors including the male sex, an age >50 years, central obesity, and a history of smoking should be considered for screening with UGI endoscopy [9,10]. However, these guidelines do not provide quantitative data to stratify the risk of BE while combining multiple risk factors. A number of risk prediction models for other diseases are widely employed to help clinicians make individualized medical decisions for their patients [11,12,13,14]; however, most of the published BE prediction models were constructed for Western populations and have yet to be verified for Asian populations [11,15,16,17,18,19,20], a relevant issue given that BE presents different patterns for Asian and Western populations [21].

Logistic regression (LR) and machine learning are two common methods for constructing risk prediction models [12,22]. LR is a classical statistical method for building prediction models, while artificial neural networks (ANNs) are state-of-the-art learning machines in the machine learning area for modeling a complex system, such as modeling, voice recognition, and image classification [23]. An ANN consists of multi-layered processing units called neurons that resemble the structure and behavior of biological neurons. The class of ANNs has the universal approximation property in the sense that they can approximate any reasonable function to any desired degree of accuracy. Traditional ANNs are referred to as “shallow” because usually only one hidden layer is employed. Recent studies have employed numerous hidden layers with different variations, which are known as deep neural networks. Srinivas et al. compared the performance between LR and ANN models for BE in patients with GERD [15]. The authors demonstrated that the ANN model was superior to the LR model, with a slightly larger area under the curve (AUC). Nevertheless, no further studies were conducted on the BE prediction models using both LR and ANN methods, especially in Asian populations.

We therefore conducted this retrospective cross-sectional study by analyzing a database of information collected during physical examinations at a health examination center in southern Taiwan. The study population underwent UGI endoscopy as part of their health examination with or without clinical indications. The aim of this study was to develop LR and ANN risk prediction models for BE in an Asian population and to comprehensively compare the predictive performance of the LR and ANN models.

## 2. Materials and Methods

### 2.1. Study Population and Data Collection

A total of 11,879 adults aged ≥20 years with or without gastrointestinal symptoms underwent UGI endoscopy during physical examinations at the Kaohsiung Veterans General Hospital, Taiwan, between 1 January 2016 and 31 December 2018. Their demographic data, history of comorbidities (including hypertension and diabetes mellitus), history of smoking (number of packs, frequency and duration), alcohol intake (number, frequency and alcohol percentage per week), exercise habits (frequency and duration per week), and GERD symptoms (e.g., heart burn, regurgitation and dysphagia) in the past three months were recorded as medical records during the pre-endoscopic examination interview. Their weight, height, and waist circumferences (WC) were measured routinely by trained examiners during health examination at our center and also recorded as medical records.

Six experienced physicians performed the endoscopic examinations at our hospital. In accordance with the American College of Gastroenterology clinical guidelines, we diagnosed BE if salmon-colored mucosa was observed extending 1 cm or more above the gastroesophageal junction, with histological confirmation of intestinal metaplasia [10]. Biopsies were obtained from four quadrants (2 cm apart) of the circumferential part of the endoscopically suspected esophageal metaplasia (ESEM). For mucosal tongues of non-circumferential ESEM, the sites and biopsies were evaluated under narrowband imaging and then decided upon by the physicians.

Of these 11,879 adults, 2233 were excluded because one had a history of esophageal cancer and 2232 had missing data in the dataset. Therefore, 9646 adults were included. The sources of the dataset were from the medical records of our hospital and the data were anonymous in the dataset. The study was approved by the Ethics Committee of the Kaohsiung Veterans General Hospital (VGHKS21-CT3-12). Participants’ consents were not required in the study because this is a retrospective study, and all data were analyzed in anonymity.

### 2.2. Model Development

We analyzed twelve candidate variables: sex, age, GERD symptoms (yes/no), a history of hypertension (yes/no), a history of diabetes mellitus (yes/no), WC, body height, body weight, BMI, history of smoking (non-smoker; ≤20 pack-years; >20 pack-years), alcohol intake (none; light-moderate drinking; heavy drinking), and regular exercise (≥3 sessions/week and ≥30 min/session).

To select the variables, we employed statsmodels (version 0.9.0, [24] in Python to construct a generalized linear model from the twelve variables (setting link function = logistic and family = binomial in statsmodels) [24] and checked each variable to determine its statistical significance (*p* < 0.05). The most significant variables will then be employed to construct the LR and ANN models for comparisons. Given the different measurement scales between the categorical and continuous variables, we employed the z-score formula to standardize all continuous variables including age, height, weight, BMI, and WC. With x as one of the input variables, the standardization process for x is performed through Equation (1):

(1) $$\hat{x}=\frac{x-\mu_{x}}{\sigma_{x}}$$

where *μ*_x_ is the mean value and *σ*_x_ is the standard deviation (SD) of the variable x. Note that in the future predicting phase, numerical type variables need to be standardized before applying the model.

**Figure 1:**
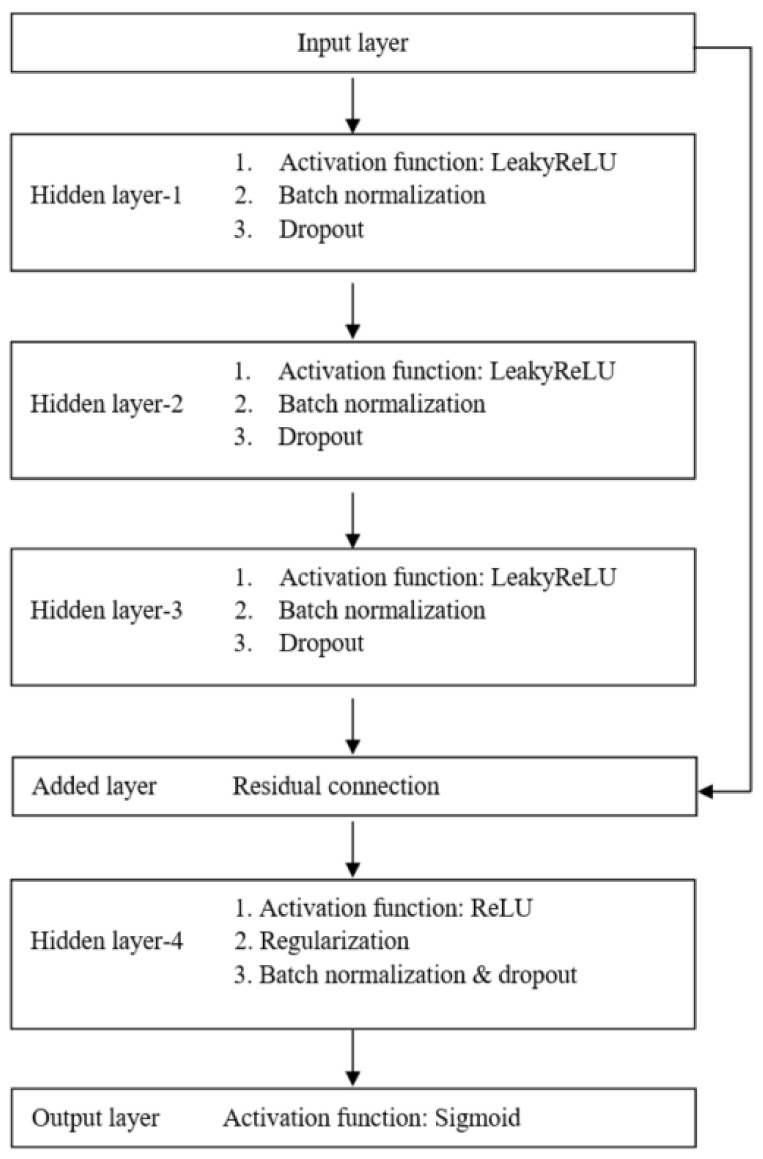
The architecture of the proposed artificial neural network model.

The LR models were built with the Python package scikit-learn (version 0.20.3) [25] and validated by stratified 10-fold cross-validation. To construct the ANN models, we employed the Python package Keras (version 2.2.4) [26]. The architecture of our proposed ANN model, presenting four hidden layers with LeakyReLU or ReLU activation functions, is shown in Figure 1. To prevent overfitting, we employed the dropout regularization mechanism [27]. Dropout is a technique which drops out the neuron connection randomly. We also adapted the batch normalization by re-centering and re-scaling the layers’ inputs for each mini batch to reduce the covariate shift between the layers in the neural network. Furthermore, the residual connection, which injects the outputs of earlier layers directly into the inputs of latter layers, is used to decrease the likelihood of representational bottleneck and vanishing gradient [28,29]. 

We employed AUC as a metric to measure model performance and validated the LR and ANN models using stratified 10-fold cross-validation. We also compared the sensitivity, specificity, and accuracy under different threshold settings of the LR and ANN models.

## 3. Results

### 3.1. Characteristics of the Subjects and Variables Selection

The simulation programs were coded using the Python programming language (version 3.5.4) running on Microsoft Windows 10 on an Intel Core i5 CPU with 8 GB of RAM. Table 1 shows the baseline characteristics of the 9646 participants, which included 5020 men and 4384 women (mean age, 50.41 years old; SD, 11.75 years), 242 of whom (2.51%) had BE. We first constructed the generalized linear model using statsmodels and analyzed the twelve variables one by one to check their statistical significance. The corresponding *p*-values are shown in Table 2. We included the four statistically significant variables (*p* < 0.05) in the successive simulations of the LR and ANN models: age (odds ratio [OR], 1.03; 95% confidence interval [CI] 1.01–1.04; *p* < 0.001), gender (OR, 1.80; 95% CI 1.15–2.82; *p* = 0.01), GERD symptoms (OR, 2.14; 95% CI 1.63–2.83; *p*< 0.001) and smoking (OR, 1.44; 95% CI 1.20–1.72; *p*< 0.001).

### 3.2. LR and ANN Models Development and Their Predictive Performance Comparisons

To better assess the trained models, we employed a stratified 10-fold cross-validation. Table 3 shows the mean performance of the stratified 10-fold cross-validation for the two models in the sampling threshold settings. With a threshold setting of 90% sensitivity, the specificity of the LR and ANN models was 31% and 20%, respectively. At 90% specificity, the sensitivity of the LR and ANN models was 30% and 28%, respectively. The point (0,1) is the best cutoff point for the perfect model (AUC = 1) on the receiver operating characteristic (ROC) curve. We therefore assumed a “closest to (0,1)” criterion to find the closest point to the top left corner (0,1) on the ROC curve, which serves as a method for finding the optimal cutoff value [30]. The values for the performance of the closest point to point (0,1) for the LR and ANN models, which are similar, are shown in Table 3.

**Figure 2:**
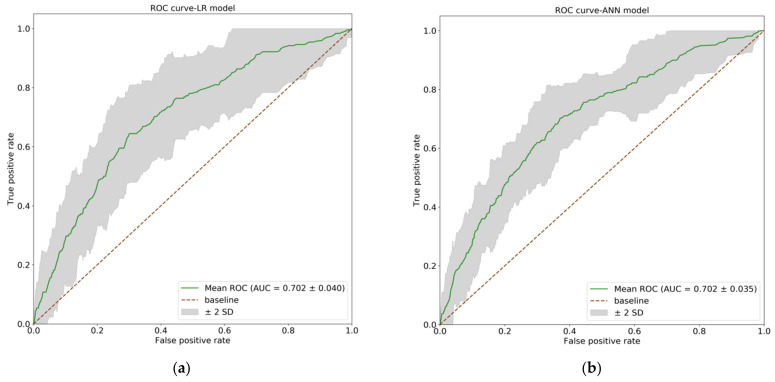
The receiver operating characteristic (ROC) curves for the logistic regression (LR) and artificial neural network (ANN) models. The green lines show the mean ROC curves, and the gray areas represent the performance within two SD around the mean ROC. (**a**) The ROC curve of LR model (AUC = 0.702, SD = 0.040); (**b**) The ROC curve of ANN model. (AUC = 0.702, SD = 0.035). AUC: area under cure; SD: standard deviation.

We computed the AUC and SD for the AUCs across folds for the LR and ANN models. Figure 2 shows the mean ROC curves (a green line) for the LR (Figure 2a) and ANN (Figure 2b) models. The gray areas represent the performance within two SD around the mean ROC. The ROC curves for the two models are very similar, with almost the same AUC (LR model: AUC, 0.702; SD, 0.040 vs. ANN model: AUC, 0.702; SD, 0.035).

### 3.3. Final Mean LR Model

We obtained the coefficients, which included the intercept of the final mean LR model, by taking the mean of the relevant coefficients across the ten folds in the cross-validation (Table 4). Assume the variables xj, j = 1,2,3,4,5 have been standardized using Equation (1) and that y denotes the estimated probability of BE, the final mean LR model through cross-validation was described using the following Equation (2):

(2) $$\begin{array}{ll} \mathrm{logit}\left(y\right) & =\ln\left(\frac{y}{1-y}\right)\\ & =-0.893+0.356x_{1}+0.697x_{2}+0.716x_{3}+0.293x_{4}+0.826x_{5} \end{array}$$

where

x_1_ is the age
x_2_ is 1 if the sex is male, otherwise 0
x_3_ is 1 if the patient has presented GERD symptoms in the past 3 months, otherwise 0
x_4_ is 1 if the patient’s cumulative smoking exposure is >0 but ≤20 pack-years, otherwise 0
x_5_ is 1 if the patient’s cumulative smoking exposure is >20 pack-years, otherwise 0

We provided the sampling threshold settings using the final mean LR model in Table 4. Based on a threshold setting of 90% sensitivity and of 90% specificity, a cutoff point was 0.33 and 0.67, respectively. Based on the “closest to (0,1)” criteria, a cutoff point was 0.52. Users can choose a suitable cutoff point according to their clinical considerations. In addition, the code of the ANN model is provided in the Supplementary Materials (see Supplementary Code S1).

## 4. Discussion

This is a large-scale study for building a risk prediction model for BE. Age, sex, GERD symptoms, and smoking were significantly associated with BE and employed to construct models. In the case of different ethnicity with different pattern of BE, our LR model had comparable performance with those of previously published models [11,15,16,17,18,19]. To our knowledge, only one published model used ANNs. Compared with that, the study had larger sample sizes and our ANN model had better performance [15]. In addition, different from previous studies, the participants in our study were not only from physicians’ referral [17,18,20] and those with subjective symptoms [15], but also from individuals without symptoms. Rubenstein et al. developed a prediction model from participants without a referral for a clinical indication; however, the study population was limited to older males (50–79 years) [11]. Our prediction models constructed by those who aged over 20 years and were not restricted by clinical indications of UGI endoscopy have the potential to be applied to the general population.

ANNs can capture nonlinear relationships between inputs (or variables) and outputs (or responses) which is considered as a powerful method of for model development. When constructing the ANNs employed in this study, we used dropout regularization techniques to avoid overfitting the training data, batch normalization to reduce the covariate shift between network layers, and residual connection to decrease the likelihood of representational bottleneck and vanishing gradient [28,29]. These methods are state-of-the-art techniques employed in deep learning and were adopted for our proposed neural network model. However, the performance of the ANN model, which was constructed by using four hidden layers, was not superior to that of the LR model in the study. The low number of significant variables for the predictive model could be a contributing factor for the result because the strength of the neural network for nonlinear mapping among these variables is limited [31].

The four most significant variables (age, sex, presence of GERD symptoms and a history of smoking), which were included as predictors in the construction of the LR and ANN models, are compatible to previous studies as risk factors [9,10]. In clinical practice, information on these four variables can be easily obtained through interviews, and the prediction process can be started with only a few variables. The stratified 10-fold cross-validation was performed in our study to minimize the variability of the models’ performance. This study demonstrated the performance characteristics of the final mean LR model for various cutoff points (Table 4). A sensitivity and specificity close to 90% having differing clinical objectives. High sensitivity is better suited to personal physical examinations to lower the proportion of undetected cases. Given the low prevalence of BE in Asian populations, a high specificity may be taken as a reference from the public health perspective. Thrift et al. indicated that, given the low risk of progression to cancer for patients with BE, the higher threshold setting for the cutoff point may be considered [16].

As a practical example for the final mean LR model, consider a 55-year-old man with GERD symptoms in the past three months but no history of smoking. After standardizing the numerical predictors in the data preprocessing step, the age variable should be adjusted to 55–50.4 ([grand mean (age)])/11.8 [SD (age)]. The formula to apply would therefore be the following (Equation (3)):

(3) $$\begin{array}{l} \mathrm{BE}\;\mathrm{probability}\\ \;=\frac{1}{1+e^{-\left(-0.893+0.356\times {\;x}_{1}+0.697\times {\;x}_{2}+0.716\times {\;x}_{3}+0.293\times {\;x}_{4}+0.826\times {\;x}_{5}\right)}}=0.66 \end{array}$$

where

$x_{1}$ is the adjusted age $=\frac{55-50.4\;\left[\mathrm{grand}\;\mathrm{mean}\;\left(\mathrm{age}\right)\right]}{11.8\;\left[\mathrm{standard}\;\mathrm{deviation}\;\left(\mathrm{age}\right)\right]}=0.39$
$x_{2}$ is 1, because the patient is male.
$x_{3\;}$ is 1, because of the GERD symptoms in the past 3 months.
$x_{4}$ is 0, because the cumulative smoking exposure is 0.
$x_{5}$ is 0, because the cumulative smoking exposure is 0.

The resulting BE probability is therefore 0.66. According to the cutoff points listed in Table 4, 0.66 is greater than 0.33, which is the cutoff point for 90% sensitivity. Endoscopic BE screening can be considered if we set cutoff point at 90% sensitivity from the personal health examination standpoint. However, the BE probability is <0.67, which is the cutoff point for 90% specificity. Endoscopic BE screening may not be suggested from the public health perspective if we set cutoff point at 90% specificity.

Our study had some limitations. First, the study enrolled participants from the center for out-of-pocket physical examinations, which may enroll individuals with a higher socioeconomic status. However, the role played by socioeconomic status in the risk of BE has not been established [9,10]. Further studies about association between socioeconomic status and BE may be needed. Second, a number of potential factors (e.g., hiatal hernia and *H. pylori* infection) were not included in this analysis [32,33], which might have improved the model. However, this information would limit the study population to those who underwent UGI endoscopy prior to the study and complicate the data collection from interviews. Third, although the stratified 10-fold cross-validation decreases the overfitting problem and provides low variability for the prediction results, external validation is still needed to confirm the model’s generalizability.

## 5. Conclusions

Both LR and ANN models exhibit good discriminative power. The ANN model did not exhibit superior performance to the LR model in the study. LR may be a preferable method for model development while low number variables employed because of its comparable performance with ANNs and interpretability in the clinical settings. However, careful interpretation of the association between independent and dependent variables is crucial due to the concerns of collapsibility and exchangeability for LR. Further studies are needed to validate the models and to evaluate the cost benefit of BE screening in different clinical settings.

## Figures and Tables

**Table 1:** Characteristics of the subjects with Barrett’s esophagus and the controls.

Variables	BE (−) *N* = 9404	BE (+) *N* = 242	Grand Mean [SD]
Mean age [SD] (years)	50.3 [11.7]	54.7 [11.4]	50.4 [11.8]
Gender			
Male	5020 (53.4%)	184 (76.0%)	
Female	4384 (46.6%)	58 (24.0%)	
Height [SD] (cm)	166.0 [8.5]	168.1 [8.1]	166.1 [8.5]
Weight [SD] (kg)	65.9 [13.2]	70.8 [12.0]	66.0 [13.1]
BMI [SD] (kg/m^2^)	23.7 [3.6]	24.9 [3.1]	23.8 [3.6]
Waist circumference [SD] (cm)	83.8 [9.8]	87.6 [8.7]	83.9 [9.8]
Hypertension	1597 (17.0%)	67 (27.7%)	
Diabetes mellitus	662 (7.0%)	31 (12.8%)	
GERD symptoms	1605 (17.1%)	81 (33.5%)	
Alcohol intake			
No	3992 (42.5%)	86 (35.5%)	
Not heavy drinking ^†^	4973 (52.9%)	142 (58.7%)	
Heavy drinking ^†^	439 (4.7%)	14 (5.8%)	
Smoking			
Non-smoker	6711 (71.4%)	125 (51.7%)	
≤20 pack-years	1790 (19.0%)	61 (25.2%)	
>20 pack-years	903 (9.6%)	56 (23.1%)	
Having Exercise habits (≥3 times/week and ≥30 mins/time)	2675 (28.4%)	77 (31.8%)	

BE: Barrett’s esophagus; BMI: Body mass index; GERD: gastroesophageal reflux disease; SD: standard deviation, ^†^ Heavy drinking was defined as 8 or more drinks a week for women and 15 or more drinks a week for men.

**Table 2:** Variables associated with Barrett’ esophagus according to multivariate analysis using generalized linear models.

Variables	Odds Ratio	95%CI	*p* Value
Age	1.03	1.01–1.04	<0.001 *
Gender (male)	1.80	1.15–2.82	0.01 *
Height (cm)	0.99	0.95–1.03	0.63
Weight (kg)	1.01	0.97–1.06	0.49
BMI (kg/m^2^)	0.99	0.87–1.13	0.91
Waist circumference (cm)	1.01	0.98–1.04	0.66
Hypertension	1.11	0.80–1.52	0.54
Diabetes mellitus	1.19	0.79–1.78	0.41
GERD symptoms	2.14	1.63–2.83	<0.001 *
Alcohol intake	0.92	0.73–1.17	0.52
Smoking	1.44	1.20–1.72	<0.001 *
Having exercise habits	0.97	0.73–1.30	0.86

BMI: Body mass index; CI: confidence interval; GERD: gastroesophageal reflux disease. * Variables with *p* < 0.05 were considered to enter into a prediction model.

**Table 3:** Mean performances of striated 10-fold cross-validation for the logistic regression and artificial neural network models using the sampling threshold settings.

Prevalence = 2.51%	LR Model			ANN Model		
Threshold Setting	Sensitivity	Specificity	Accuracy	Sensitivity	Specificity	Accuracy
Sensitivity~90%	0.90	0.31	0.32	0.90	0.20	0.22
Specificity~90%	0.30	0.90	0.88	0.28	0.90	0.88
The Closest to (0,1) Criteria	0.65	0.68	0.68	0.63	0.65	0.65

ANN: artificial neural network; LR: logistic regression.

**Table 4:** Coefficients of the final mean logistic regression model and its performance using the sampling threshold settings.

Coefficients and Adjusted OR		Performances of Whole Data Input in Final Mean LR Model				
Variables	Adjusted OR	Threshold Setting	Cutoff Point	Sensitivity	Specificity	Accuracy
Age [SD]	1.43	Specificity~90%	0.67	0.30	0.90	0.88
Gender(male) [SD]	2.01	Specificity~80%	0.58	0.46	0.80	0.80
GERD [SD]	2.05	Sensitivity~90%	0.33	0.90	0.32	0.33
Smoking		Sensitivity~80%	0.46	0.80	0.46	0.40
Non smokers	1	The Closest to (0,1) Criteria	0.52	0.65	0.69	0.70
≤20 pack-years [SD]	1.34					
>20 pack-years [SD]	2.28					
Intercept						

LR: logistic regression; OR: odds ratio; SD: standard deviation; GERD: gastroesophageal reflux disease.

## Data Availability

Data is contained within the Supplementary Materials. The data presented in this study are available in Supplementary Data S2.

## Supplementary Materials

The following are available online at https://www.mdpi.com/article/10.3390/ijerph18105332/s1, Code S1: The code for ANN model is provided in the file. Data S2: The dataset used in this study is provided in the file.
