# Supplementary material for: Risk Prediction of Barrett’s Esophagus in a Taiwanese Health Examination Center Based on Regression Models
Source: Int J Environ Res Public Health. 2021 May 17;18(10):5332. doi: 10.3390/ijerph18105332 (PMC8157048; doi:10.3390/ijerph18105332)
Supplement: Supplementary file 1 [file ijerph-18-05332-s001.zip › Code S1.pdf]

```
import os
mywd='E:\\Google  雲端硬碟\\AI\\Barrets\\dataset\\'
os.chdir(mywd)
os.getcwd()

import numpy as np
import matplotlib.pyplot as plt
from keras.models import Sequential, Model, load_model
from keras.layers import add, Conv1D,MaxPooling1D,Flatten
from keras.layers import Input, Dense, Dropout, Activation, BatchNormalization
from keras.layers import add, regularizers, LeakyReLU
from sklearn.metrics import confusion_matrix, f1_score,
precision_recall_fscore_support
from sklearn.metrics import average_precision_score,
matthews_corrcoef,precision_recall_curve
from sklearn import ensemble, preprocessing, metrics
from collections import Counter
from numpy import genfromtxt
import pandas as pd

#data=np.genfromtxt("barrets_HW_lab4.txt", delimiter = ",",skip_header=1)
dataPD=pd.read_csv("barrets_2journal.csv")
print(dataPD.head())

##transfer data to numpy for better calculation
data=dataPD.to_numpy()
X_index=[0,1,2,3,5,6,7,8,9,10,11,12]
X = data[:,X_index]
Y = np.array(data[:,4], dtype = int)
print(X.shape)
print(Y.shape)
print(np.unique(Y))
print(np.bincount(Y))

## GLM
import statsmodels.api as sm
X_t_test=np.copy(X)
X_t_test = sm.add_constant(X_t_test)
```

```
model = sm.GLM(Y,X_t_test,family=sm.families.Binomial(sm.families.links.logit)).fit()

model.summary()
## x1:P>|t|  那個值就是 P value

## select p<0.05 variables for candidates[age, gender, GERD, PY]
X_select_index=[2,3,8,10]
X_select=X[:,X_select_index]
print(X[:5])
print(X_select[:5])

## hanfle OneHotEncoder for PY
Smokingindex=[3]
Smoking=X_select[:,Smokingindex]
from sklearn.preprocessing import OneHotEncoder
enc = OneHotEncoder()
sm_one=enc.fit(Smoking)
sm_array=sm_one.transform(Smoking).toarray()
print(sm_array[-20:])
print(Smoking[-20:])
X_selONEhot=np.concatenate((X_select[:,:-1],sm_array[:,1:]),axis=1)
print(X_select[-20:])
print(X_selONEhot[-20:])

##standarize for age
standardize = lambda x:(x - np.mean(x, axis = 0)) / np.std(x, axis = 0, ddof = 1)
X_standarize=np.copy(X_selONEhot)
X_standarize[:,0]=standardize(X_standarize[:,0])
print(X_standarize[-10:])

def build_model(alphaN,neuron1, neuron2, neuron3, dropoutRATE):
    Input_data=Input(shape=(ann_input_shape,))
    y1=Dense(units=neuron1)(Input_data)
    LR1=LeakyReLU(alpha=alphaN)(y1)
    Batch_y1=BatchNormalization()(LR1)
    d1=Dropout(rate=dropoutRATE)(Batch_y1)
    y2=Dense(units=neuron2)(d1)
    LR2=LeakyReLU(alpha=alphaN)(y2)
```

```
        Batch_y2=BatchNormalization()(LR2)
        d2=Dropout(rate=dropoutRATE)(Batch_y2)
        y3=Dense(units=neuron1)(d2)
        LR3=LeakyReLU(alpha=alphaN)(y3)
        Batch_y3=BatchNormalization()(LR3)
        d3=Dropout(rate=dropoutRATE)(Batch_y3)
        y_add=add([d3,Batch_y1])
        Batch_add=BatchNormalization()(y_add)
        d_add=Dropout(rate=dropoutRATE)(Batch_add)
        y4=Dense(units=neuron3,
activation='relu',kernel_regularizer=regularizers.l1_l2(l1=0.5, l2=0.5))(d_add)
        Batch_y4=BatchNormalization()(y4)
        d4=Dropout(rate=dropoutRATE)(Batch_y4)
        output_layer=Dense(units=1, activation='sigmoid')(d4)
        model = Model(inputs = Input_data, outputs = output_layer)
        return model


import tensorflow as tf
import keras.backend as K
def f1_loss(y_true, y_pred):
        tp = K.sum(K.cast(y_true*y_pred, 'float'), axis=0)
        tn = K.sum(K.cast((1-y_true)*(1-y_pred), 'float'), axis=0)
        fp = K.sum(K.cast((1-y_true)*y_pred, 'float'), axis=0)
        fn = K.sum(K.cast(y_true*(1-y_pred), 'float'), axis=0)

        p = tp / (tp + fp + K.epsilon())
        r = tp / (tp + fn + K.epsilon())

        f1 = 2*p*r / (p+r+K.epsilon())
        f1 = tf.where(tf.is_nan(f1), tf.zeros_like(f1), f1)
        return 1 - K.mean(f1)

# calculate fbeta score for multi-class/label classification
def fbeta(y_true, y_pred, beta=2):
# clip predictions
        y_pred = K.clip(y_pred, 0, 1)
# calculate elements
```

```
        tp = K.sum(K.round(K.clip(y_true * y_pred, 0, 1)), axis=0)
        fp = K.sum(K.round(K.clip(y_pred - y_true, 0, 1)), axis=0)
        fn = K.sum(K.round(K.clip(y_true - y_pred, 0, 1)), axis=0)
# calculate precision
        p = tp / (tp + fp + K.epsilon())
# calculate recall
        r = tp / (tp + fn + K.epsilon())
# calculate fbeta, averaged across each class
        bb = beta ** 2
        fbeta_score = K.mean((1 + bb) * (p * r) / (bb * p + r + K.epsilon()))
        return fbeta_score

from keras.callbacks import EarlyStopping
from keras.callbacks import ModelCheckpoint
from keras.callbacks import ReduceLROnPlateau
# callbacks:

es=EarlyStopping(monitor='loss', patience=2, verbose=0, mode = 'min')

rp=ReduceLROnPlateau(monitor='loss',factor=0.07,patience=1,verbose=0,mode='aut
o',epsilon=0.1,cooldown=1,min_lr=1e-8)
callbacks=[es,rp]

cv_lgr_pred=np.zeros((10,970))
cv_lgr_coef=np.zeros((10,5))
cv_lgr_intercept=[]
cv_ANN_pred=np.zeros((10,970))
cv_number=[]
cv_true=np.zeros((10,970))
CV_time=1

from sklearn.linear_model import LogisticRegression
from sklearn.model_selection import RepeatedStratifiedKFold
from keras import optimizers
from keras.models import load_model

rskf = RepeatedStratifiedKFold(n_splits=10, n_repeats=1,random_state=8)
for train_index, test_index in rskf.split(X=X_standarize, y=Y):
```

```
        X_RSKF_train, X_RSKF_test = X_standarize[train_index],
X_standarize[test_index]
        y_RSKF_train, y_RSKF_test = Y[train_index], Y[test_index]
        cv_number.append(len(y_RSKF_test))
        LGR = LogisticRegression(random_state=0, solver='liblinear',
                                                  class_weight='balanced').fit(X_RSKF_train,
y_RSKF_train)
        cv_lgr_coef[CV_time-1]=LGR.coef_
        cv_lgr_intercept.append(LGR.intercept_[0])
        pred_LGR=LGR.predict_proba(X_RSKF_test)
        pred_LGR=pred_LGR[:,1]
        for i, el in enumerate(pred_LGR):
                cv_lgr_pred[CV_time-1,i]=el

        for i, el in enumerate(y_RSKF_test):
                cv_true[CV_time-1,i]=el

        ann_input_shape=len(X_RSKF_train[0])
        neuron1=ann_input_shape*9
        neuron2=ann_input_shape*6
        neuron3=ann_input_shape
        MoDel=build_model(alphaN=0.1,neuron1=15, neuron2=5, neuron3=5,
dropoutRATE=0.2)
        opt = optimizers.Adam(lr=0.005)
        MoDel.compile(loss =f1_loss, optimizer = opt, metrics = ['accuracy'])
        history=MoDel.fit(X_RSKF_train, y_RSKF_train, epochs = 100, batch_size = 200,
verbose = 0
                                      ,class_weight={0:1, 1:120},shuffle=True,callbacks
=callbacks)
        MoDel.save('E:\\Google 雲端硬碟
\\AI\\Barrets\\Barrets2journal\\model_%d.h5'%(CV_time))
        pred_ANN=MoDel.predict(X_RSKF_test)
        pred_ANN=pred_ANN[:,0]
        for i, el in enumerate(pred_ANN):
                cv_ANN_pred[CV_time-1,i]=el
        K.clear_session()
        del MoDel
```

```
        print(CV_time)
        CV_time+=1

AUC_lgr=[]
fpr_lgr_list=[]
tpr_lgr_list=[]

for i in range(10):
        fpr_lgr, tpr_lgr, th_lgr = metrics.roc_curve(cv_true[i,0:cv_number[i]],
cv_lgr_pred[i,0:cv_number[i]])
        AUC_lgr.append(metrics.auc(fpr_lgr, tpr_lgr))
        fpr_lgr_list.append(fpr_lgr)
        tpr_lgr_list.append(tpr_lgr)

#################LGR    ROC curve###########################
tprs_lgr=[]
mean_fpr_lgr=np.linspace(0,1,1000)
for j in range(10):
        tprs_lgr.append(np.interp(mean_fpr_lgr, fpr_lgr_list[j], tpr_lgr_list[j]))

mean_tpr_lgr=np.mean(tprs_lgr, axis=0)
mean_tpr_lgr[-1]=1.0
mean_auc_lgr=metrics.auc(mean_fpr_lgr, mean_tpr_lgr)
std_auc_lgr=np.std(AUC_lgr,ddof = 1 )
std_tpr_lgr = np.std(tprs_lgr, axis=0,ddof = 1)
tprs_upper_lgr = np.minimum(mean_tpr_lgr + 2 * std_tpr_lgr, 1) ##because
np.minimum requires 2 input arrays!
tprs_lower_lgr = np.maximum(mean_tpr_lgr - 2 * std_tpr_lgr, 0) ##broadcast 1/0
array

import matplotlib.pyplot as plt
fig=plt.figure()

plt.plot(mean_fpr_lgr, mean_tpr_lgr, color='green',
                        label=r'Mean ROC (AUC = %0.3f $\pm$ %0.3f)' % (mean_auc_lgr,
std_auc_lgr), linewidth=2.0, alpha=0.80)
plt.fill_between(mean_fpr_lgr, tprs_lower_lgr, tprs_upper_lgr, color='grey',
alpha=0.33, label=r'$\pm$ 2 std. dev.')
```

```
plt.plot([0,1],[0,1],color='saddlebrown', lw=2, linestyle='--', label='baseline')
plt.xlim([0.0,1.0])
plt.ylim([0.0,1.05])
plt.xlabel('False positive rate')
plt.ylabel('True positive rate')
plt.title('ROC curve-LR model')
plt.legend(loc='lower right')
##https://stackoverflow.com/questions/12444716/how-do-i-set-the-figure-title-and-axes-labels-font-size-in-matplotlib?rq=1
import matplotlib.pylab as pylab
params = {'legend.fontsize': 'xx-large',
          'figure.figsize': (12, 12),
         'axes.labelsize': 'xx-large',
         'axes.titlesize':'xx-large',
         'xtick.labelsize':'xx-large',
         'ytick.labelsize':'xx-large'}
pylab.rcParams.update(params)
plt.show()


#################ANN    ROC curve###########################
AUC_ann=[]
fpr_ann_list=[]
tpr_ann_list=[]

for i in range(10):
    fpr_ann, tpr_ann, th_ann = metrics.roc_curve(cv_true[i,0:cv_number[i]], cv_ANN_pred[i,0:cv_number[i]])
    AUC_ann.append(metrics.auc(fpr_ann, tpr_ann))
    fpr_ann_list.append(fpr_ann)
    tpr_ann_list.append(tpr_ann)

tprs_ann=[]
mean_fpr_ann=np.linspace(0,1,1000)
for j in range(10):
    tprs_ann.append(np.interp(mean_fpr_ann, fpr_ann_list[j], tpr_ann_list[j]))

mean_tpr_ann=np.mean(tprs_ann, axis=0)
```

```
mean_tpr_ann[-1]=1.0
mean_auc_ann=metrics.auc(mean_fpr_ann, mean_tpr_ann)
std_auc_ann=np.std(AUC_ann,ddof = 1 )
std_tpr_ann = np.std(tprs_ann, axis=0,ddof = 1)
tprs_upper_ann = np.minimum(mean_tpr_ann + 2 * std_tpr_ann, 1) ##because
np.minimum requires 2 input arrays!
tprs_lower_ann = np.maximum(mean_tpr_ann - 2 * std_tpr_ann, 0) ##broadcast 1/0
array

import matplotlib.pyplot as plt
fig=plt.figure()

plt.plot(mean_fpr_ann, mean_tpr_ann, color='green',
                    label=r'Mean ROC (AUC = %0.3f $\pm$ %0.3f)' % (mean_auc_ann,
std_auc_ann), linewidth=2.0, alpha=0.80)
plt.fill_between(mean_fpr_ann, tprs_lower_ann, tprs_upper_ann, color='grey',
alpha=0.33, label=r'$\pm$ 2 std. dev.')
plt.plot([0,1],[0,1],color='saddlebrown', lw=2, linestyle='--', label='baseline')
plt.xlim([0.0,1.0])
plt.ylim([0.0,1.05])
plt.xlabel('False positive rate')
plt.ylabel('True positive rate')
plt.title('ROC curve-ANN model')
plt.legend(loc='lower right')
##https://stackoverflow.com/questions/12444716/how-do-i-set-the-figure-title-and-
axes-labels-font-size-in-matplotlib?rq=1
import matplotlib.pylab as pylab
params = {'legend.fontsize': 'xx-large',
              'figure.figsize': (12, 12),
             'axes.labelsize': 'xx-large',
             'axes.titlesize':'xx-large',
             'xtick.labelsize':'xx-large',
             'ytick.labelsize':'xx-large'}
pylab.rcParams.update(params)
plt.show()

#################ANN    ROC curve###########################
AUC_ann=[]
```

```
fpr_ann_list=[]
tpr_ann_list=[]

for i in range(10):
	fpr_ann, tpr_ann, th_ann = metrics.roc_curve(cv_true[i,0:cv_number[i]],
cv_ANN_pred[i,0:cv_number[i]])
	AUC_ann.append(metrics.auc(fpr_ann, tpr_ann))
	fpr_ann_list.append(fpr_ann)
	tpr_ann_list.append(tpr_ann)

tprs_ann=[]
mean_fpr_ann=np.linspace(0,1,1000)
for j in range(10):
	tprs_ann.append(np.interp(mean_fpr_ann, fpr_ann_list[j], tpr_ann_list[j]))

mean_tpr_ann=np.mean(tprs_ann, axis=0)
mean_tpr_ann[-1]=1.0
mean_auc_ann=metrics.auc(mean_fpr_ann, mean_tpr_ann)
std_auc_ann=np.std(AUC_ann,ddof = 1 )
std_tpr_ann = np.std(tprs_ann, axis=0,ddof = 1)
tprs_upper_ann = np.minimum(mean_tpr_ann + 2 * std_tpr_ann, 1) ##because
np.minimum requires 2 input arrays!
tprs_lower_ann = np.maximum(mean_tpr_ann - 2 * std_tpr_ann, 0) ##broadcast 1/0
array

import matplotlib.pyplot as plt
fig=plt.figure()

plt.plot(mean_fpr_ann, mean_tpr_ann, color='green',
			label=r'Mean ROC (AUC = %0.3f $\pm$ %0.3f)' % (mean_auc_ann,
std_auc_ann), linewidth=2.0, alpha=0.80)
plt.fill_between(mean_fpr_ann, tprs_lower_ann, tprs_upper_ann, color='grey',
alpha=0.33, label=r'$\pm$ 2 std. dev.')
plt.plot([0,1],[0,1],color='saddlebrown', lw=2, linestyle='--', label='baseline')
plt.xlim([0.0,1.0])
plt.ylim([0.0,1.05])
plt.xlabel('False positive rate')
plt.ylabel('True positive rate')
```

```
plt.title('ROC curve-ANN model')
plt.legend(loc='lower right')
##https://stackoverflow.com/questions/12444716/how-do-i-set-the-figure-title-and-axes-labels-font-size-in-matplotlib?rq=1
import matplotlib.pylab as pylab
params = {'legend.fontsize': 'xx-large',
           'figure.figsize': (12, 12),
          'axes.labelsize': 'xx-large',
          'axes.titlesize':'xx-large',
          'xtick.labelsize':'xx-large',
          'ytick.labelsize':'xx-large'}
pylab.rcParams.update(params)
plt.show()

##get mean LGR
cv_lgr_coef.shape
cv_lgr_intercept
lgr_mean_coef=np.mean(cv_lgr_coef, axis=0)
lgr_std_coef=np.std(cv_lgr_coef, axis=0,ddof = 1)
lgr_mean_intercept=np.mean(cv_lgr_intercept)
lgr_std_intercept=np.std(cv_lgr_intercept,ddof=1)
np.exp(lgr_mean_coef)


def lgr_mean_pred(X_train):

lgr_mean_pred=1/(1+np.exp(-(lgr_mean_intercept+lgr_mean_coef[0]*X_train[:,0]
                            +lgr_mean_coef[1]*X_train[:,1]
                            +lgr_mean_coef[2]*X_train[:,2]
                            +lgr_mean_coef[3]*X_train[:,3]
                            +lgr_mean_coef[4]*X_train[:,4]
                           )))
    return lgr_mean_pred

mean_pred_lgr_all=lgr_mean_pred(X_standarize)
fpr_lgr_all, tpr_lgr_all, th_lgr_all = metrics.roc_curve(Y,mean_pred_lgr_all)
mean_lgr_all_AUC=metrics.auc(fpr_lgr_all, tpr_lgr_all)
```

```
##find threshold
def threshold_show(y_test, Y_predict):
        Threshold_list = []
        Accuracy_list = []
        Sensitivity_list = []
        Specificity_list = []
        PPV_list = []
        NPV_list = []
        F1_list = []
        for t in range(10001):
                Threshold = t/10000
                Threshold_list.append(Threshold)
                Y_pred_class = [0 if item < Threshold else 1 for item in Y_predict]
                tn, fp, fn, tp = confusion_matrix(y_test, Y_pred_class).ravel()
                Accuracy = (tp+tn)/(tn+fp+fn+tp)
                Sensitivity = tp / (tp+fn)
                Specificity = tn / (fp+tn)
                PPV = tp/ (tp+fp)
                NPV = tn / (tn+fn)
                F1 = 2*PPV*Sensitivity / (PPV+Sensitivity)
                Accuracy_list.append(Accuracy)
                Sensitivity_list.append(Sensitivity)
                Specificity_list.append(Specificity)
                PPV_list.append(PPV)
                NPV_list.append(NPV)
                F1_list.append(F1)
        return
Threshold_list,Accuracy_list,Sensitivity_list,Specificity_list,PPV_list,NPV_list,F1_list


###LGR
y_test_lgr=np.array([])
Y_predict_lgr=np.array([])

for i in range(10):
        y_test_lgr=np.concatenate([y_test_lgr,cv_true[i,0:cv_number[i]]])
        Y_predict_lgr=np.concatenate([Y_predict_lgr,cv_lgr_pred[i,0:cv_number[i]]])
```

```
lgr_threshold=threshold_show(y_test=y_test_lgr, Y_predict=Y_predict_lgr)
LGR_dict={'Threshold_list':lgr_threshold[0],
          'Accuracy_list':lgr_threshold[1],
          'Sensitivity_list':lgr_threshold[2],
          'Specificity_list':lgr_threshold[3],
          'PPV_list':lgr_threshold[4],
          'NPV_list':lgr_threshold[5],
          'F1_list':lgr_threshold[6]}
LGR_dict2DF=pd.DataFrame.from_dict(LGR_dict)
LGR_dict2DF.to_csv('D:\\Google 雲端硬碟
\\AI\\Barrets\\result\\new_lgr_threshold2.csv')

####ANN threshold#####
y_test_ann=np.array([])
Y_predict_ann=np.array([])

for i in range(10):
    y_test_ann=np.concatenate([y_test_ann,cv_true[i,0:cv_number[i]]])

Y_predict_ann=np.concatenate([Y_predict_ann,cv_ANN_pred[i,0:cv_number[i]]])

ann_threshold=threshold_show(y_test=y_test_ann, Y_predict=Y_predict_ann)
ANN_dict={'Threshold_list':ann_threshold[0],
          'Accuracy_list':ann_threshold[1],
          'Sensitivity_list':ann_threshold[2],
          'Specificity_list':ann_threshold[3],
          'PPV_list':ann_threshold[4],
          'NPV_list':ann_threshold[5],
          'F1_list':ann_threshold[6]}
ANN_dict2DF=pd.DataFrame.from_dict(ANN_dict)
ANN_dict2DF.to_csv('D:\\Google 雲端硬碟
\\AI\\Barrets\\result\\mean_ann_threshold2.csv')

##LGR mean    mean_pred_lgr_all


mean_lgr_threshold=threshold_show(y_test=Y, Y_predict=mean_pred_lgr_all)
```

```
mean_LGR_dict={'Threshold_list':mean_lgr_threshold[0],
               'Accuracy_list':mean_lgr_threshold[1],
               'Sensitivity_list':mean_lgr_threshold[2],
               'Specificity_list':mean_lgr_threshold[3],
               'PPV_list':mean_lgr_threshold[4],
               'NPV_list':mean_lgr_threshold[5],
               'F1_list':mean_lgr_threshold[6],
               'MCC_list':mean_lgr_threshold[7]}
mean_LGR_dict2DF=pd.DataFrame.from_dict(mean_LGR_dict)
mean_LGR_dict2DF.to_csv('E:\\Google 雲端硬碟
\\AI\\Barrets\\Barrets2journal\\mean_lgr_threshold.csv')
```
